# The Effect of Single Versus Group μCT on the Detection of Trabecular and Cortical Disease Phenotypes in Mouse Bones

**DOI:** 10.1002/jbm4.10473

**Published:** 2021-03-05

**Authors:** Rachel Kohler, Carli A Tastad, Alexander J Stacy, Elizabeth A Swallow, Corinne E Metzger, Matthew R Allen, Joseph M Wallace

**Affiliations:** ^1^ Weldon School of Biomedical Engineering department of Purdue University West Lafayette IN USA; ^2^ Department of Biomedical Engineering Indiana University Purdue University of Indianapolis Indianapolis IN USA; ^3^ Department of Anatomy and Cell Biology Indiana University School of Medicine Indianapolis IN USA; ^4^ Division of Nephrology, Department of Medicine Indiana University School of Medicine Indianapolis IN USA; ^5^ Roudebush Veterans Administration Medical Center Indianapolis IN USA

**Keywords:** BONE MICRO‐COMPUTED TOMOGRAPHY (μCT), CHRONIC KIDNEY DISEASE, OSTEOGENESIS IMPERFECTA, PRECLINICAL STUDIES

## Abstract

Micro‐computed tomography is a critical assessment tool for bone‐related preclinical research, especially in murine models. To expedite the scanning process, researchers often image multiple bones simultaneously; however, it is unknown if this impacts scan quality and alters the ability to detect differences between experimental groups. The purpose of this study was to assess the effect of multibone scanning on detecting disease‐induced changes in bone microarchitecture and mineral density by group scanning two murine models with known skeletal defects: the *Col1a2* ^G610C/+^ model of osteogenesis imperfecta and an adenine‐induced model of chronic kidney disease. Adult male femurs were scanned individually and in random groups of three and eight in a Bruker Skyscan 1172 and 1176, respectively, then assessed for standard trabecular and cortical bone measures. Although scanning methodology altered raw values, with trabecular microarchitecture values more affected than cortical properties, a disease phenotype was still detectable in both group and solo scans. However, tissue mineral density in both trabecular and cortical bone was significantly impacted by group versus solo scanning. Researchers may be able to use small groupings in a single μCT scan to expedite preclinical analyses when the overall bone phenotype is large to decrease costs and increase speed of discoveries; however the details of scanning (single or group) should always be reported. © 2021 The Authors. *JBMR Plus* published by Wiley Periodicals LLC on behalf of American Society for Bone and Mineral Research.

## Introduction

μCT is a nondestructive ex vivo imaging technique that is a critical assessment tool for bone‐related research. Importantly, μCT has resolution capabilities able to adequately assess mouse bone, the most typical animal model used for preclinical research. The determination of the microarchitecture of trabecular bone is a key use of μCT. This technology has been used to show the differences in trabecular bone volume in models such as hindlimb‐unloading,^(^ ^1^, ^2^ ^)^ spaceflight,^(^ ^3^, ^4^ ^)^ ovariectomy,^(^ ^5^ ^)^ exercise and mechanical‐loading studies,^(^ ^6^ ^)^ aging,^(^ ^7^ ^)^ and drug treatment,^(^ ^8^ ^)^ as well as diseases such as chronic kidney disease (CKD)^(^ ^9^ ^)^ and osteogenesis imperfecta (OI).^(^ ^10^ ^)^ Similarly, μCT has also been used to detect changes in cortical bone morphology including cortical area, cortical thickness, and cortical porosity.^(^ ^7^, ^9^, ^11^, ^12^, ^13^ ^)^ μCT scans are widely used to calculate BMD and tissue mineral density (TMD),^(^ ^14^ ^)^ achieving greater precision and reproducibility than other common analytical tools such as DEXA^(^ ^15^ ^)^ or pQCT.

Although μCT scans are highly useful, they can be time‐consuming and expensive. Depending on the device used and the scan parameters set, individual scan times can range from 5 minutes to well over an hour. As a single study can often require 10 to 100 of scans, this can become quite costly in usage fees and personnel hours. Additionally, these systems are often shared; thus they have limited hours available for each investigator, which can further constrain the progression of experiments.

One expense‐ and time‐saving measure that can expedite the scanning process is scanning multiple bones simultaneously. This group‐scanning method is especially attractive with mouse bones, as their small size allows multiple bones to fit within a single‐scan view. A potential downside is the variability in how the bones pass between the source and detector during rotation, perhaps altering the attenuation. Although guidelines for μCT analysis have already been established for most other settings,^(^ ^14^ ^)^ it is largely unknown if scanning multiple bones simultaneously alters the ability to detect differences among treatments. If it does, this would be an important detail for researchers to document in methods to increase rigor and reproducibility. The purpose of this study was to assess the effect of multibone scanning on detecting disease‐induced changes in bone microarchitecture and BMD. We hypothesized that scanning multiple bones at a time would attenuate differences between groups leading to reduced ability to detect group differences compared with single‐bone scans.

## Materials and Methods

### Specimens

Femurs from male mice from two different disease models with known skeletal alterations were used. The rationale behind using two different models was to help increase the generalizability of the results beyond a single data set. The first set of bones came from the *Col1a2* ^G610C/+^ (G610C) model of OI. WT (*n* = 8) and heterozygous (G610C; *n* = 8) mice bred on a C57BL/6J background strain originally from Jackson Laboratories were group housed (two to three per cage) in an institutionally approved facility. Mice were euthanized at 16 weeks of age via carbon dioxide asphyxiation and cervical dislocation, after which left femurs were immediately harvested, stripped of soft tissue, and stored in saline‐soaked gauze at −20°C until scanning. Animal procedures were approved by the Indiana University School of Science Institutional Animal Use and Care Committee prior to the initiation of experimental protocols.

A second set of samples came from an adenine‐induced chronic kidney disease (CKD) model that increases bone resorption, which results in high cortical porosity. Male C57Bl/6J mice (*n* = 16) were ordered from Jackson Laboratories at 15 weeks of age and group housed (four per cage) at an institutionally approved animal facility. Adenine‐induced CKD mice (*n* = 8) were placed on an adenine diet from 16 weeks of age to 22 weeks, and then returned to the control diet with a maintained calcium and phosphorus ratio for another 5 weeks for a total of 11 weeks since diet initiation. The adenine diet consisted of 0.2% adenine added to a diet of purified casein‐based feed with adjusted calcium and phosphorus (0.9% phosphorus, 0.6% calcium) as previously described.^(^ ^9^ ^)^ A set of age‐matched controls (*n* = 8) were fed the casein‐based control diet with an altered calcium:phosphorus ratio for the entire 11‐week period. At study end point, all mice were anesthetized via vaporized inhaled isoflurane and euthanized via exsanguination. Right femora were harvested, stripped of soft tissue, and stored in saline‐soaked gauze at −20°C until scanning.

### Fixture design and μCT

**FIG 1:**
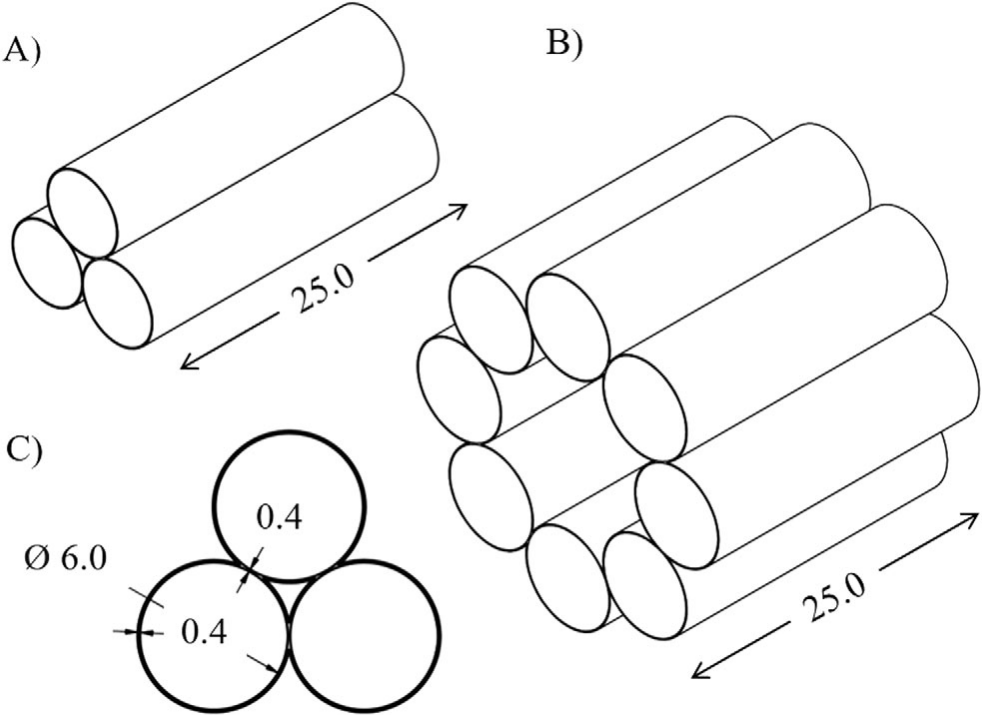
Schematics of custom specimen holders. (*A*) Small holder with three‐bone capacity used in the Bruker Skyscan 1172. (*B*) Larger holder with eight‐bone capacity used in the Skyscan 1176. (*C*) Inner dimensions of each specimen “tube” and wall thickness. All units are in mm.

μCT analysis was first conducted on a Bruker Skyscan 1176. A custom‐designed fixture (3D printout of FormLabs clear V4 photoreactive resin) was used to secure the bones in equidistantly spaced chambers arranged in a ring (Fig. 1). Notches marked on the holder provided geometric markers to keep track of individual bones. For all scans, thawed bones were wrapped in Parafilm to maintain tissue hydration. Femurs were first scanned individually within the custom holder, and then scanned again in groups of eight, with four bones from each set (CKD and OI) randomly placed in each scanning group. All scans were conducted at a nominal voxel size of 9 μm, using a 0.5‐mm Al filter (V = 60 kV, I = 167 μA) with a 0.7°angle increment and two frames averaged. Hydroxyapatite‐mimicking phantoms (0.25 and 0.75 g/cm^3^ Ca HA) were scanned individually in the holders at each scanning session to allow calculation of TMD.

**FIG 2:**
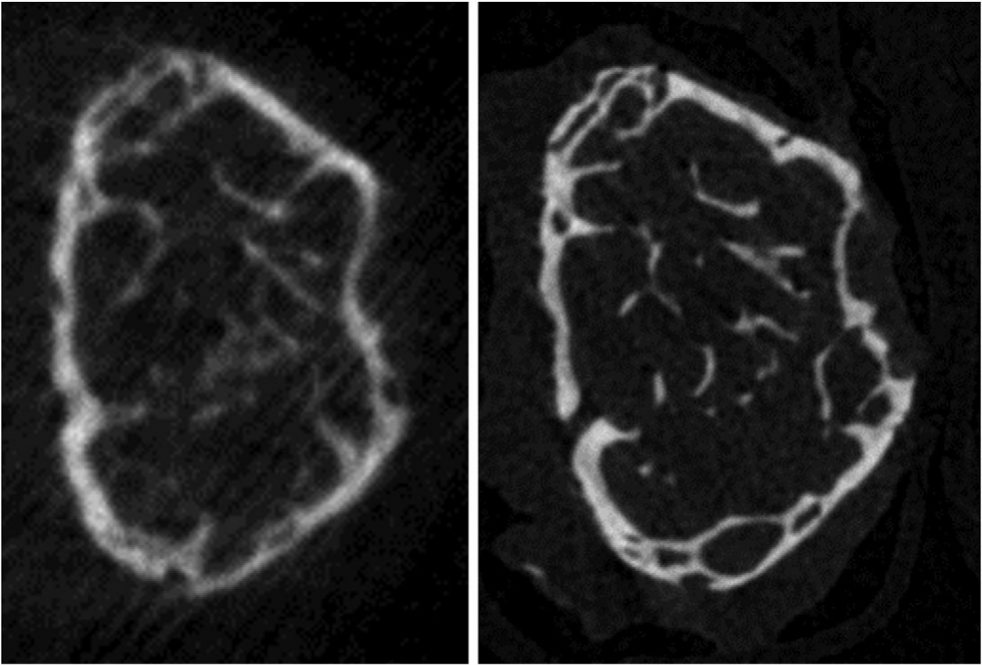
System variance and effect on image quality. Shown are trabecular cross sections of the same adenine‐treated femur scanned on the Bruker Skyscan 1176 (*left*) and the Skyscan 1172 (*right*), at approximately the same position. Nominal settings were identical for both scans, but system‐specific differences in factors such as signal‐to‐noise ratio and geometric magnification result in vastly different image qualities.

Subsets of specimens from both disease models were next scanned on a Bruker Skyscan 1172 to assess the effects of multibone scanning on cortical porosity, which is difficult to resolve using the Skyscan 1176 at our typical scan settings (Fig. 2). Because of the smaller scanning aperture and smaller field of view, a second custom fixture was printed that could hold three bones (Fig. 1). Scanning was performed using six bones randomly chosen from each set of samples. Thawed bones were again wrapped in Parafilm and scanned first individually within the custom holder, then in random groups of three. The nominal scan settings were identical to those used on the Skyscan 1176.

### μCT analysis

Image analysis was performed with Bruker's custom Skyscan software. Scans for each bone were reconstructed with NRecon. All reconstruction parameters were identical for individual and group scans: 2 for smoothing (Gaussian), ring artifact correction of 8, and beam hardening correction at 20. Reconstructed images were then rotated to ensure consistent cross‐sectional analysis (Data Viewer) and saved in a transaxial orientation. A 1‐mm trabecular region of interest (ROI) was selected in the distal metaphysis starting at the most proximal portion of the distal femur growth plate and extending proximally. ROIs of internal trabecular bone were isolated from this initial selection of whole‐bone slices using a batch‐processing CT analyzer (CTAn) code. A 0.1‐mm cortical ROI was taken at the approximate midpoint of the diaphysis. The trabecular microarchitecture within trabecular ROIs was assessed using CTAn, while cortical properties were computed with a custom MatLab (MathWorks) program described previously,^(^ ^16^ ^)^ with a threshold value of a 70 used to distinguish bone in both cases. Cortical porosity values from the Skyscan 1172 adenine scans were measured as the void space between the periosteal and endocortical bone from an average of five slices, 2‐mm proximal to the end of the trabecular region of the distal femur metaphysis.

### Statistical analyses

First, a repeated measure two‐way ANOVA (disease‐by‐scan type) was performed separately for adenine and G610C data sets for each μCT system to assess the effect of scanning methodology and disease model on computed variables. Main effects of scan type and disease are reported (*p* > 0.05). In the case of a significant interaction term, main effects were disregarded and a Sidak multiple‐comparison post hoc analysis was completed. A second analysis was undertaken to compare the ability to detect a disease phenotype with different scan configurations and model a real‐world application more directly without the increased complexity of a multifactorial ANOVA. A two‐tailed Student *t* test was performed within bone sets (control vs adenine, WT vs G610C), and within the same scanning methodology (solo‐scanned separately and group‐scanned separately). To compare the strength of the ability of each scan type to detect a disease phenotype, effect sizes from the *t* tests were reported using Cohen D methodology. Data from the secondary *t* test analyses are only reported in Table 3. All other graphs and tables report the results of the repeated measures two‐way ANOVA. GraphPad PRISM 8.4.1 (GraphPad Software) was used to perform all statistical tests. All data are represented as mean ± SD.

## Results

### Trabecular microarchitecture

**FIG 3:**
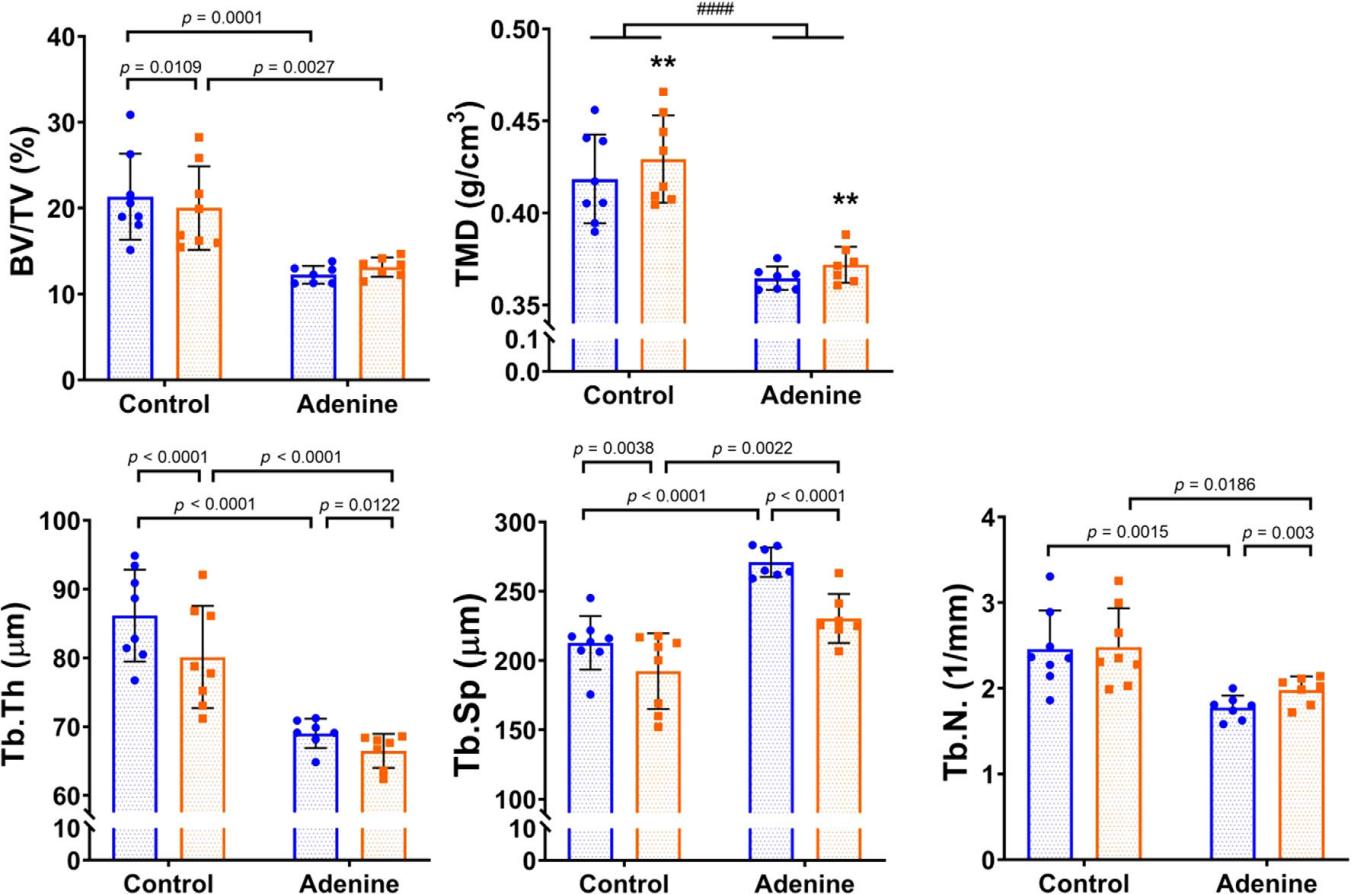
Adenine trabecular properties calculated from Skyscan 1176 scans. Blue circles indicate solo‐scan data; orange squares indicate group scan data. All statistics are from the repeated measures 2 × 2 ANOVA (disease‐by‐scan type). Asterisk “*” denotes main effects of scan type (**p ≤ 0.01), pound sign “#” denotes main effect of disease (####*p* < 0.0001). *p* Values displayed are from post hoc analyses from measures with a significant interaction effect. BV/TV = bone volume tissue volume; Tb.N = trabecular number; Tb.Sp = trabecular spacing; Tb.Th = trabecular thickness; TMD = trabecular tissue mineral density.

When comparing group versus solo scanning on the Skyscan 1176 system, an interaction effect between disease and scan types was present in four of the five trabecular properties for adenine mice compared with controls (% bone volume [BV/TV] *p* = 0.002, trabecular thickness [Tb.Th] *p* = 0.0063, trabecular spacing [Tb.Sp] *p* = 0.0226, trabecular number [Tb.N] *p* = 0.0042). Only trabecular TMD lacked a disease‐by‐scan‐type interaction (*p* = 0.4583), and instead had main effects of disease (*p* < 0.0001) and scan type (*p* = 0.001). For all properties with significant interactions, post hoc analyses showed that though adenine and control group averages were significantly different when bones were scanned individually (solo‐scanned control vs solo‐scanned adenine), disease differences in trabecular parameters were still detected when bones were group scanned (Fig. 3). For the WT versus G610C bones, there was a main effect of scan type on Tb.Th (*p* = 0.017) and an effect of genotype on Tb.Sp (*p* = 0.0005) and Tb.N (*p* = 0.0109), but no interaction effects (see Table 1). In the secondary analysis, the *t‐*test and effect‐size results (for control vs adenine) were inconsistent between group and solo scanning for most trabecular bone properties (see Table 3). Effect‐size values of WT versus G610C bones were equally mixed, with no clear pattern for either single or group scanning.

**Table 1:** Trabecular Microarchitecture

No. specimens	Control		Adenine		*p* Value		
	n = 1	n = 8	n = 1	n = 8	Disease	Scan type	Disease * scan type
Adenine 1176							
BV/TV (%)	21.3 ± 5	20 ± 4.9	12.2 ± 1	13.2 ± 1.1	**0.001**	0.5088	**0.002**
Tb.Th (μm)	86.2 ± 6.7	80.1 ± 7.4	69 ± 2.1	66.5 ± 2.5	**<0.0001**	**<0.0001**	**0.0063**
Tb.Sp (μm)	213 ± 20	192 ± 30	271 ± 10	230 ± 20	**0.0002**	**<0.0001**	**0.0226**
Tb.N (1/mm)	2.46 ± 0.45	2.48 ± 0.45	1.77 ± 0.14	1.98 ± 0.16	**0.0052**	**0.0008**	**0.0042**
TMD (g/cm^3^ HA)	0.42 ± 0.024	0.43 ± 0.024	0.36 ± 0.006	0.37 ± 0.01	**<0.0001**	**0.0013**	0.4583

No. specimens	WT		G610C		*p* Value		
	n = 1	n = 8	n = 1	n = 8	Genotype	Scan type	Genotype * scan type
G610C 1176							
BV/TV (%)	31.3 ± 7.2	29 ± 5.5	23.5 ± 8.1	22.9 ± 10.1	0.0916	0.0853	0.2987
Tb.Th (μm)	83.7 ± 9.4	79.15 ± 6.5	78.8 ± 12.6	78 ± 14.1	0.5885	**0.0169**	0.0831
Tb.Sp (μm)	159 ± 20	155 ± 20	198 ± 20	195 ± 20	**0.0005**	0.4325	0.9202
Tb.N (1/mm)	3.71 ± 0.52	3.65 ± 0.5	2.92 ± 0.51	2.84 ± 0.71	**0.0109**	0.3328	0.9076
TMD (g/cm^3^ HA)	0.39 ± 0.019	0.39 ± 0.019	0.39 ± 0.029	0.39 ± 0.03	0.9411	0.2027	0.2162

No. specimens	Control		Adenine		*p* Value		
	n = 1	n = 3	n = 1	n = 3	Disease	Scan type	Disease * scan type
Adenine 1172							
BV/TV (%)	15.4 ± 5.12	13.5 ± 2.55	10.1 ± 0.67	9.9 ± 0.85	**0.0021**	0.5911	0.7056
Tb.Th (μm)	67 ± 6	66 ± 5	58 ± 2	57 ± 2	**0.0014**	0.4503	0.9934
Tb.Sp (μm)	216 ± 34	232 ± 12	284 ± 9	285 ± 9	**<0.0001**	0.4976	0.5162
Tb.N (1/mm)	2.3 ± 0.62	2 ± 0.28	1.8 ± 0.12	1.7 ± 0.15	**0.0134**	0.6301	0.6795
TMD (g/cm^3^ HA)	0.86 ± 0.06	0.8 ± 0.04	0.84 ± 0.02	0.84 ± 0.02	0.0637	**<0.0001**	0.2875

No. specimens	WT		G610C		*p* Value		
	n = 1	n = 3	n = 1	n = 3	Genotype	Scan type	Genotype * scan type
G610C 1172							
BV/TV (%)	20 ± 4.87	19.4 ± 4.82	15.3 ± 4.52	14.3 ± 4.19	0.0946	**<0.0001**	0.1113
Tb.Th (μm)	61 ± 5	60 ± 5	57 ± 7	56 ± 6	0.2707	**0.0102**	0.7404
Tb.Sp (μm)	180 ± 15	181 ± 15	197 ± 7	200 ± 6	**0.0213**	**0.0008**	**0.0274**
Tb.N (1/mm)	3.3 ± 0.52	3.2 ± 0.56	2.7 ± 0.45	2.5 ± 0.46	**0.0475**	**<0.0001**	**0.0194**
TMD (g/cm^3^ HA)	0.73 ± 0.03	0.7 ± 0.02	0.73 ± 0.03	0.69 ± 0.03	0.9778	**<0.0001**	**0.0112**

Parameters of the G610C and adenine disease models assessed by μCT (Bruker Skyscan 1172 and 1176). Values presented as group means ± SD. All statistics are from the repeated measures 2 × 2 ANOVA (disease‐by‐scan type). The *p* values from the repeated measures 2‐way ANOVA are presented for disease/genotype, scan type, and interaction effects. Statistically significant values (*p* < 0.05) are emphasized in bold.

Abbreviations: BV/TV = % bone volume; Tb.Th = trabecular thickness; Tb.Sp = trabecular spacing; Tb.N = trabecular number; TMD = trabecular tissue mineral density.

**FIG 4:**
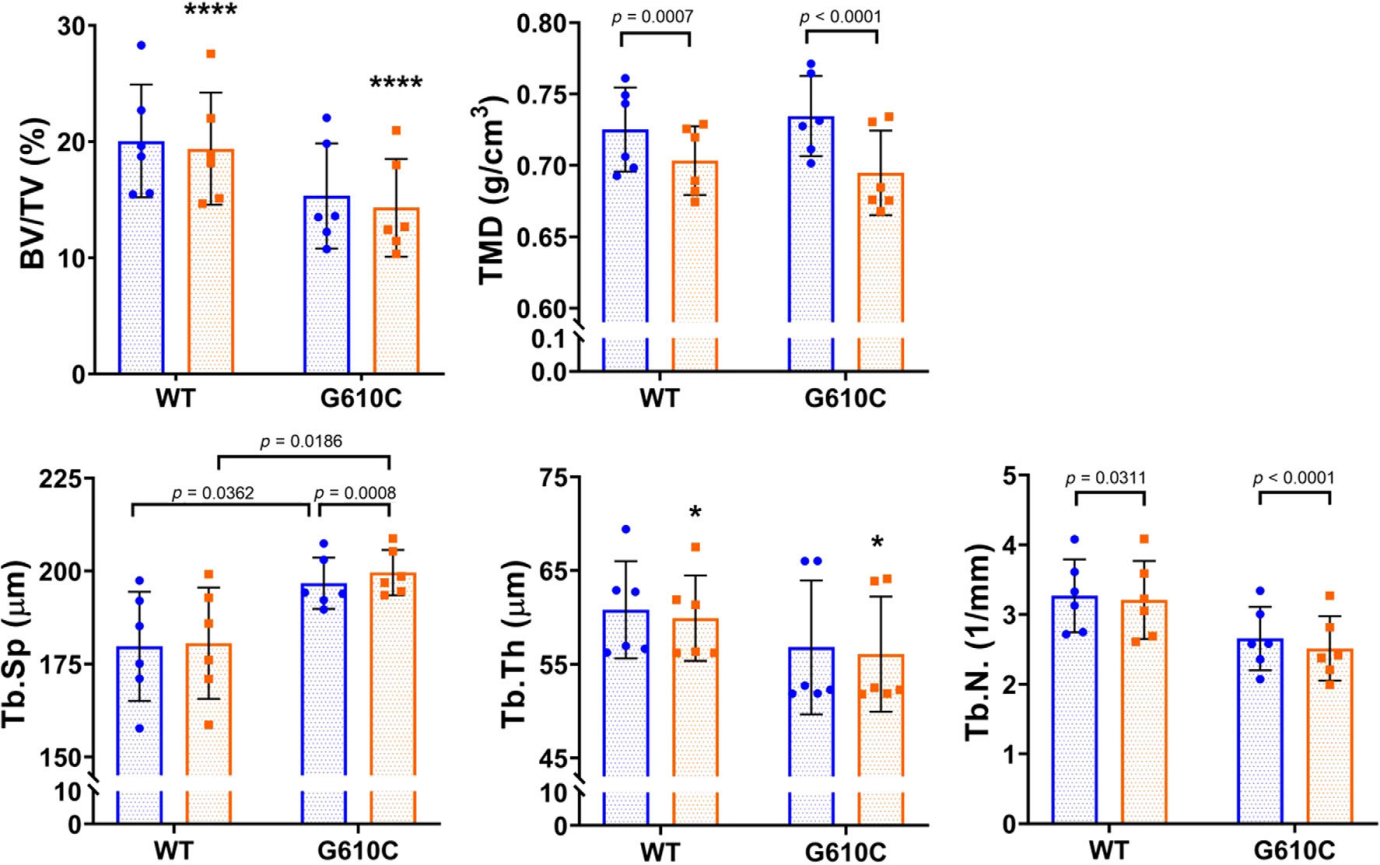
G610C trabecular properties calculated from Skyscan 1172 scans. Blue circles indicate solo scan data; orange squares indicate group scan data. All statistics are from the repeated measures 2 × 2 ANOVA (disease‐by‐scan type). Asterisk “*” denotes main effects of scan type (*p ≤ 0.05, ****p < 0.0001). *p* Values displayed are from post hoc analyses from measures with a significant interaction effect. BV/TV = bone volume tissue volume; Tb.N = trabecular number; Tb.Sp = trabecular spacing; Tb.Th = trabecular thickness; TMD = trabecular tissue mineral density.

When comparing group with solo scanning on the Skyscan 1172 system, there were no interactions between disease and scan type for trabecular properties of adenine mice compared with controls (Table 1). Although numerous main effects of disease were noted, main effects of scan type were found only for TMD (*p* < 0.0001), and there were no significant interactions. For the WT versus G610C bones, there were interaction effects for Tb.Sp (*p* = 0.0274), Tb.N (*p* = 0.0194), and TMD (*p* = 0.0112), as well as main effects of scan type for BV/TV (*p* < 0.0001) and Tb.Th (*p* = 0.01). Post hoc analyses showed that though group scanning affected individual values as compared with solo scanning, group averages remained unchanged and the genotype trends were still detected (Fig. 4). The secondary analysis supported this, with *t‐*test results and effect‐size values similar between the two scan modalities for both the adenine versus control and WT versus G610C bones (Table 3).

### Cortical microarchitecture

When comparing group with solo scanning on the Skyscan 1176 system, an interaction effect between disease and scan type for the adenine set was only present at the femur midshaft for minimum moment of inertia (*p* = 0.0016), where group‐scanned control bones displayed higher values compared with solo‐scanned control bones. There were significant main effects of scan type on total cross‐sectional area (*p* = 0.0266), cortical area (*p* = 0.003), cortical thickness (*p* = 0.007), and cortical TMD (*p* = 0.022; Table 2 **)**. There were no interaction effects between genotype and scan type for any cortical parameters between WT and G610C bones (Table 2). Within the G610C data set, a main effect of scan type was present for many parameters as shown in Table 2. In the secondary analysis comparing control with adenine and WT with G610C, the effect size of each cortical property was similar between group scans and solo scans for all properties (Table 3).

**Table 2:** Cortical Microarchitecture

No. specimens	Control		Adenine		*p* Value		
	n = 1	n = 8	n = 1	n = 8	Disease	Scan type	Disease * scan type
Adenine 1176							
Total CSA (mm^2^)	2.1 ± 0.14	2 ± 0.14	1.9 ± 0.13	1.92 ± 0.13	0.0837	**0.0266**	0.3275
Marrow area (mm^2^)	1.1 ± 0.12	1.1 ± 0.11	1.2 ± 0.14	1.18 ± 0.14	0.2575	0.3111	0.416
Cortical area (mm^2^)	0.95 ± 0.06	0.94 ± 0.06	0.75 ± 0.03	0.74 ± 0.03	**<0.0001**	**0.0033**	0.0891
Cortical thickness (mm)	0.22 ± 0.01	0.22 ± 0.01	0.17 ± 0.01	0.17 ± 0.01	**<0.0001**	**0.007**	0.0514
Periosteal BS (mm)	5.9 ± 0.18	5.9 ± 0.2	5.7 ± 0.18	5.7 ± 0.19	0.0573	0.1018	0.9368
Endocortical BS (mm)	4.7 ± 0.2	4.7 ± 0.23	4.8 ± 0.15	4.8 ± 0.13	0.3	0.2843	0.5288
Imax (mm^4^)	0.35 ± 0.039	0.35 ± 0.041	0.27 ± 0.026	0.26 ± 0.024	**0.0001**	0.1017	0.7642
Imin (mm^4^)	0.16 ± 0.02	0.15 ± 0.02	0.12 ± 0.01	0.12 ± 0.01	**0.001**	**0.0069**	**0.0016**
Section modulus (mm^3^)	0.25 ± 0.03	0.25 ± 0.02	0.2 ± 0.017	0.2 ± 0.018	**0.0004**	0.9444	0.9366
TMD (g/cm^3^ HA)	1.58 ± 0.03	1.61 ± 0.03	1.45 ± 0.03	1.46 ± 0.03	**<0.0001**	**0.0219**	0.0546

No. specimens	WT		G610C		*p* Value		
	n = 1	n = 8	n = 1	n = 8	Genotype	Scan type	Genotype * scan type
G610C 1176							
Total CSA (mm^2^)	1.9 ± 0.13	1.9 ± 0.12	1.7 ± 0.2	1.68 ± 0.2	**0.0110**	**0.0140**	0.6848
Marrow area (mm^2^)	0.98 ± 0.1	1 ± 0.08	0.79 ± 0.08	0.8 ± 0.09	**0.0006**	**0.0063**	0.631
Cortical area (mm^2^)	0.96 ± 0.07	0.93 ± 0.06	0.91 ± 0.16	0.88 ± 0.16	0.4293	**0.0003**	0.8117
Cortical thickness (mm)	0.23 ± 0.02	0.22 ± 0.01	0.23 ± 0.03	0.23 ± 0.03	0.6827	**0.0005**	0.7136
Periosteal BS (mm)	5.7 ± 0.15	5.6 ± 0.13	5.3 ± 0.29	5.3 ± 0.28	**0.0101**	**0.0074**	0.9911
Endocortical BS (mm)	4.4 ± 0.12	4.4 ± 0.12	4.1 ± 0.33	4.1 ± 0.29	**0.0179**	0.8636	0.5058
Imax (mm^4^)	0.31 ± 0.031	0.3 ± 0.03	0.27 ± 0.07	0.26 ± 0.071	0.1038	**<0.0001**	0.6751
Imin (mm^4^)	0.16 ± 0.02	0.15 ± 0.02	0.12 ± 0.04	0.12 ± 0.04	**0.0417**	**0.0119**	0.9639
Section modulus (mm^3^)	0.24 ± 0.03	0.24 ± 0.02	0.21 ± 0.043	0.2 ± 0.043	0.0587	**0.0126**	0.0765
TMD (g/cm^3^ HA)	1.53 ± 0.02	1.55 ± 0.03	1.6 ± 0.06	1.61 ± 0.06	**0.0039**	0.284	0.8677

No. specimens	Control		Adenine		*p* Value		
	n = 1	n = 3	n = 1	n = 3	Disease	Scan type	Disease * scan type
Adenine 1172							
Total CSA (mm^2^)	1.98 ± 0.15	1.98 ± 0.15	1.95 ± 0.09	1.92 ± 0.09	0.627	**0.0002**	0.7852
Marrow area (mm^2^)	1.1 ± 0.11	1.1 ± 0.11	1.2 ± 0.08	1.2 ± 0.1	0.0906	**0.0088**	0.2734
Cortical area (mm^2^)	0.9 ± 0.05	0.89 ± 0.05	0.74 ± 0.03	0.74 ± 0.04	**0.0002**	0.5605	0.2128
Cortical thickness (mm)	0.21 ± 0.01	0.21 ± 0.01	0.17 ± 0.01	0.17 ± 0.01	**<0.0001**	0.9353	0.1381
Periosteal BS (mm)	5.7 ± 0.23	5.7 ± 0.2	5.7 ± 0.14	5.7 ± 0.12	0.7522	**0.0463**	0.0958
Endocortical BS (mm)	4.6 ± 0.18	4.6 ± 0.21	4.8 ± 0.11	4.9 ± 0.19	0.1225	0.2244	0.2703
Imax (mm^4^)	0.32 ± 0.05	0.32 ± 0.04	0.27 ± 0.02	0.27 ± 0.02	**0.0362**	0.7309	0.4244
Imin (mm^4^)	0.15 ± 0.02	0.15 ± 0.02	0.13 ± 0.01	0.12 ± 0.01	0.0577	**0.0013**	0.7881
Section modulus (mm^3^)	0.24 ± 0.02	0.24 ± 0.02	0.21 ± 0.01	0.2 ± 0.01	**0.0038**	**0.0105**	0.3338
TMD (g/cm^3^ HA)	1.52 ± 0.02	1.41 ± 0.02	1.46 ± 0.04	1.35 ± 0.03	**0.0029**	**<0.0001**	0.9035
% Cortical porosity	0.312 ± 0.08	0.322 ± 0.020	3.628 ± 2.00	3.282 ± 2.07	**0.007**	0.3624	0.336

No. specimens	WT		G610C		*p* Value		
	n = 1	n = 3	n = 1	n = 3	Genotype	Scan type	Genotype * scan type
G610C 1172							
Total CSA (mm^2^)	1.9 ± 0.11	1.9 ± 0.11	1.58 ± 0.17	1.59 ± 0.17	**0.0039**	0.3697	0.1017
Marrow area (mm^2^)	1.01 ± 0.06	1.01 ± 0.06	0.78 ± 0.1	0.78 ± 0.1	**0.0008**	0.1844	0.9242
Cortical area (mm^2^)	0.89 ± 0.07	0.89 ± 0.07	0.81 ± 0.12	0.81 ± 0.12	0.164	0.1541	0.5289
Cortical thickness (mm)	0.21 ± 0.01	0.21 ± 0.01	0.21 ± 0.03	0.21 ± 0.03	0.9843	0.2061	0.5312
Periosteal BS (mm)	5.6 ± 0.13	5.6 ± 0.13	5.1 ± 0.24	5.2 ± 0.24	**0.0034**	0.2083	0.1676
Endocortical BS (mm)	4.4 ± 0.16	4.4 ± 0.17	4 ± 0.35	4 ± 0.35	**0.0187**	0.1442	0.4895
Imax (mm^4^)	0.29 ± 0.03	0.29 ± 0.03	0.22 ± 0.05	0.22 ± 0.05	**0.0193**	0.6305	0.6188
Imin (mm^4^)	0.15 ± 0.02	0.15 ± 0.02	0.11 ± 0.03	0.11 ± 0.03	**0.0158**	0.1176	0.1319
Section modulus (mm^3^)	0.24 ± 0.03	0.24 ± 0.03	0.18 ± 0.04	0.18 ± 0.04	**0.0103**	0.4111	0.5307
TMD (g/cm^3^ HA)	1.22 ± 0.02	1.2 ± 0.01	1.29 ± 0.02	1.24 ± 0.02	**0.0001**	**<0.0001**	**0.0491**

Parameters of the G610C and adenine disease models assessed by μCT (Skyscan 1172 and 1176). Values presented as group means ± SD. All statistics are from the repeated measures 2 × 2 ANOVA (disease‐by‐scan type). The *p* values from the repeated measures 2‐way ANOVA are presented for disease/genotype, scan type, and interaction effects. Statistically significant values (*p* < 0.05) are emphasized in bold.

Abbreviations: BS = bone surface; CSA = total cross‐sectional area; I = moment of inertia; TMD = cortical tissue mineral density.

**Table 3:** Secondary Analysis

No. specimens		Student *t* test (*p* value)								Cohen D effect size			
		1172 Data				1176 Data				1172 Data		1176 Data	
		n = 1		n = 3		n = 1		n = 8		n = 1	n = 3	n = 1	n = 8
Adenine (CKD)													
Cortical	Total CSA	0.63		0.46		0.08		0.09		0.29	0.45	0.94	0.92
	Marrow area	0.07		0.13		0.25		0.27		1.25	0.94	0.60	0.58
	Cortical area	1.7E‐04	****	1.0E‐04	****	4.6E‐07	****	5.4E‐07	****	3.64	3.57	4.39	4.33
	Cortical thickness	1.3E‐05	****	5.3E‐05	****	2.5E‐06	****	2.5E‐06	****	5.31	3.87	3.80	3.80
	Periosteal BS	0.64		0.70		0.06		0.06		0.28	0.23	1.03	1.00
	Endocortical BS	0.17		0.10		0.36		0.27		0.87	1.04	0.47	0.58
	Imax	0.04	*	0.02	*	1.7E‐04	****	1.7E‐04	****	1.41	1.66	2.53	2.54
	Imin	0.06		0.03	*	0.001	***	0.001	**	1.27	1.49	2.18	1.97
	Section modulus	0.005	**	0.002	**	0.001	***	2.2E‐04	****	2.21	2.47	2.07	2.47
	TMD	0.02	*	0.002	**	2.3E‐06	****	9.5E‐08	****	1.79	2.43	3.82	4.99
Trabecular	BV/TV	0.03	*	0.01	**	0.13		0.67		1.44	1.88	0.87	0.21
	Tb.Th	0.01	**	0.003	**	0.43		0.02	*	2.05	2.26	0.42	1.39
	Tb.Sp	0.001	***	6.7E‐06	****	0.60		0.31		2.76	4.92	0.28	0.53
	Tb.N	0.07		0.04	*	0.36		0.36		1.18	1.36	0.50	0.47
	BMD	0.002	**	0.003	**	0.88		0.03	*	2.49	2.29	0.08	1.28
	TMD	0.41		0.01	*	0.46		0.003	**	0.50	1.82	0.40	1.83

No. specimens		Student *t* test (*p* value)								Cohen D effect size			
		1172 Data				1176 Data				1172 Data		1176 Data	
		n = 1		n = 3		n = 1		n = 8		n = 1	n = 3	n = 1	n = 8
G610C (OI)													
Cortical	Total CSA	0.01	**	0.004	**	0.01	*	0.01	*	2.17	2.15	1.45	1.47
	Marrow area	0.001	***	0.001	***	0.001	***	4.6E‐04	****	2.77	2.71	2.07	2.27
	Cortical area	0.20		0.16		0.42		0.44		0.86	0.87	0.42	0.39
	Cortical thickness	0.99		0.96		0.72		0.65		0.00	0.03	0.18	0.23
	Periosteal BS	0.01	**	0.004	**	0.01	*	0.01	*	2.25	2.15	1.45	1.51
	Endocortical BS	0.02	*	0.02	*	0.04	*	0.01	*	1.60	1.63	1.12	1.46
	Imax	0.03	*	0.02	*	0.10		0.11		1.59	1.62	0.88	0.86
	Imin	0.02	*	0.02	*	0.04	*	0.04	*	1.68	1.67	1.10	1.14
	Section modulus	0.02	*	0.01	**	0.09		0.04	*	1.77	1.87	0.90	1.15
	TMD	4.1E‐04	****	0.001	***	0.01	*	0.01	*	3.49	2.75	1.43	1.51
Trabecular	BV/TV	0.08		0.08		0.34		0.11		1.01	1.13	0.50	0.85
	Tb.Th	0.25		0.25		0.74		0.25		0.64	0.71	0.17	0.60
	Tb.Sp	0.01	**	0.02	*	0.52		0.34		1.48	1.66	0.33	0.50
	Tb.N	0.03	*	0.04	*	0.40		0.16		1.25	1.35	0.44	0.74
	BMD	0.08		0.11		0.73		0.02	*	1.10	1.00	0.18	1.36
	TMD	0.82		0.59		0.54		0.78		0.33	0.32	0.32	0.14
									**KEY:**	No effect	Little	Medium	Large

Results of Student *t* test between control and adenine, and separately WT and G610C. *p* Values and effect sizes are reported. The differences in effect size are shaded from light to dark highlighting magnitude.

Abbreviations: BS = bone surface; BV/TV = % bone volume; CKD = chronic kidney disease; CSA = total cross‐sectional area; Imax = maximum moment of inertia; Imin = minimum moment of inertia; OI = osteogenesis imperfecta; Tb.Th = trabecular thickness; Tb.Sp = trabecular spacing; Tb.N = trabecular number; TMD = trabecular tissue mineral density.

*p ≤ 0.05, **p ≤ 0.01, ***p ≤ 0.001, ****p < 0.0001.

**FIG 5:**
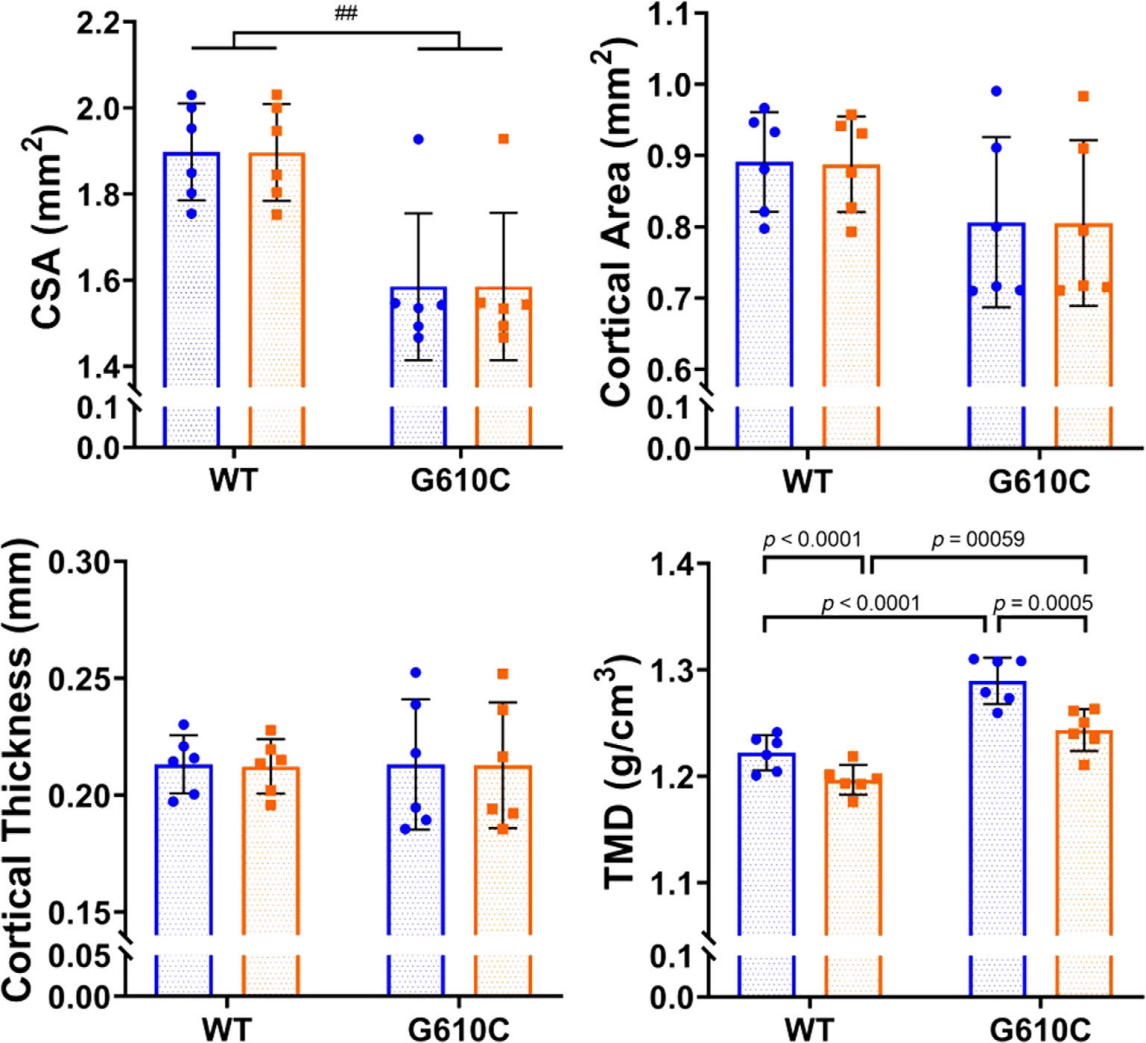
G610C cortical properties calculated from Bruker Skyscan 1172 scans. Blue circles indicate solo scan data; orange squares indicate group scan data. All statistics are from the repeated measures 2 × 2 ANOVA (disease‐by‐scan type). Pound sign “#” denotes the main effect of disease (##*p* ≤ 0.01). *p* Values displayed are from post hoc analyses from measures with a significant interaction effect. CSA = total cross‐sectional area; TMD = cortical tissue mineral density.

When comparing group with solo scanning on the Skyscan 1172 system, the main effects of scan type were present for many parameters in the adenine versus control group as noted in Table 2. Cortical porosity of adenine‐treated mice was not affected by the scan type (*p* = 0.3624; Table 2). For the WT versus G610C group, there was an interaction effect for TMD (*p* = 0.0491), with post hoc analysis showing that group scanning reduced group averages, although phenotype differences were still detected (Fig. 5). Trends and magnitudes of effect sizes between control versus adenine and WT versus G610C within each scan type were similar for all properties (Table 3).

## Discussion

μCT analysis is primarily used to determine if a given intervention or genotype produces differences in geometry, microarchitecture, or TMD of bone compared with a set of control specimens. Although reproducible data are desirable, differences both within and across laboratories in methodology and measuring systems can affect the output data; hence, the behavior of overall trends is more informative and enables better comparison with other studies. The results of this study show that scanning methodology, whether by group or individual scans, had minimal effects on trends within data sets, especially for cortical properties.

It is important that researchers recognize that group scanning affects system output, although it appears that in most cases this does not compromise the ability to detect the existence of a severe disease phenotype. Because of this, it is important that scanning methodology (individual or group scans) is reported in methods when describing the scanning procedure. Further consideration and exploration of other possible caveats of multibone scanning and their impacts on bone outcomes, such as the impacts of studying different numbers of bones per scan, would be important before using such methodology in a study. We speculate that the number of bones scanned together may impact output data because more bones have a greater potential for interference between the x‐ray source and the detector. However, this interference effect would need to be shown experimentally.

The results here show that for animal models with severe bone phenotypes, multibone scanning does not affect the ability to detect differences in bone phenotype, especially in cortical parameters. However, it is important to note that the results show the greatest interaction between scan type and disease in trabecular microarchitecture parameters. This interaction was particularly pronounced with the Skyscan 1176 data, potentially based on the lower image quality of 9‐μm scans on this system. Group scanning compounded the limitations of lower‐quality output images, inhibiting the ability to detect differences in trabecular microarchitecture. Additionally, TMD in both trabecular and cortical bone was significantly impacted by group versus solo scanning. Therefore, careful consideration of group scanning should be taken if TMD is a primary end point.

There are limitations to the scope of this study. First, group‐scanned bones cannot be directly compared between the two systems: The Skyscan 1176 bones were scanned in groups of eight, whereas the Skyscan 1172 bones were scanned in groups of three because of the smaller field of view. It is possible that there are a minimal number of bones that can be scanned before group scanning has an effect, but this cannot be determined from this study. Second, we speculate that the scan‐based differences reported here with male femurs would extend to other bones, ages, sexes, etc., but the magnitude may vary based on the amount of bone material and TMD of a given sample. Researchers using a different type of sample set are advised to perform their own test scans before beginning a full analysis. Third, although the goal of this study was not to compare output from different machines, there are differences in the data obtained from the same samples on the two different systems, particularly with the trabecular data. For example, absolute values gathered on the Skyscan 1172 were higher by 20%–50%, 15%–30%, and 5%–25% for trabecular BV/TV, Tb.Th , and Tb.N , respectively, as compared with the Skyscan 1176 data. These results agree with previous work by Verdelis et al, that trabecular morphometry can have high intersystem variability.^(^ ^17^ ^)^ This highlights the importance of investigators selecting the machine that best suits their needs for each data set, optimizing scan settings, and consistently reporting all these variables for reproducibility and the ability to compare data across different studies. Finally, newer systems may perform better or worse than the Bruker Skyscan 1176 and 1172 used here; regardless, the burden of proof remains on the researcher to show that they have minimized interference by testing and reporting scan setup.

In conclusion, scanning bones in groups may be a viable way to increase throughput and reduce the costs of μCT analysis based on the samples and the primary outcomes measured. However, the exact method and number of bones per scan should be reported to allow for reproducibility. Researchers should exercise caution when measuring microscale features such as trabecular microarchitecture or quantifying unknown or subtle phenotypes; the interference of other bones in a group scan may obscure small‐scale differences.

## AUTHOR CONTRIBUTIONS

**Rachel Kohler:** Data curation; formal analysis; visualization; writing‐original draft; writing‐review & editing. **Carli Tastad:** Formal analysis; writing‐original draft. **Alexander Stacy:** Data curation. **Elizabeth Swallow:** Writing‐review & editing. **Corinne Metzger:** Formal analysis; validation; writing‐review & editing. **Matt Allen:** Conceptualization; project administration; resources; writing‐review & editing. **Joseph Wallace:** Conceptualization; project administration; resources; supervision; writing‐review & editing.

## Conflict of Interest

The authors have nothing to disclose.

### Peer Review

The peer review history for this article is available at https://publons.com/publon/10.1002/jbm4.10473.

## Data Availability

Data is available upon request.
